# Supplementary material for: Rapid Microfluidic Mixer Based on Ferrofluid and Integrated Microscale NdFeB-PDMS Magnet
Source: Micromachines (Basel). 2019 Dec 25;11(1):29. doi: 10.3390/mi11010029 (PMC7019818; doi:10.3390/mi11010029)
Supplement: Supplementary file 1 [file micromachines-11-00029-s001.pdf]

Supplementary Material

Rapid Microfluidic Mixer Based on Ferrofluid and Integrated Microscale NdFeB-PDMS Magnet

Ran Zhou [1,*], Athira N Surendran[1], Marcel Mejulu[1] and Yang Lin[2]

[1] Department of Mechanical and Civil Engineering, Purdue University Northwest, Hammond, IN 46321, USA;
[2] Department of Mechanical and Industrial Engineering, University of Illinois at Chicago, Chicago, IL 60607, USA.
* Correspondence: zhou970@pnw.edu

CFD simulation modeling details:

The modeling strategy consists of two steps: 1) simulation of the DC magnetic field using FEMM based on the micro-magneto configurations; 2) import the magnetic flux intensity field from the FEMM simulation results into the CFD model and perform the coupled laminar flow and MHD simulations for the mixture. A 2-D steady-state CFD model is developed to simulate the mixing process, with the following configuration:

- Both water and ferrofluid adopt constant molecular viscosities and densities, but the viscosity and density of the mixture vary based on the local ferrofluid concentration.
- The computational mesh consists of 0.2 million quadrilateral cells for the 2-D model.
- Ferrofluid concentration is treated as a passive scalar solved via a user-defined scalar (UDS).
- Magnetic forces are incorporated into the momentum equations as source terms through the user-defined functions (UDF)
- For boundary conditions (B.C.):
    - Velocity inlet B.C. is used for both water and ferrofluid;
    - Pressure outlet B.C. is adopted at channel outlet, with a total pressure of 1 atm;
    - No-slip B.C. is used for all the channel walls (zero velocities and zero gradient for pressure and UDS).
